# Transcatheter aortic valve replacement via femoral artery for aortic regurgitation post-LVAD: a case report

**DOI:** 10.3389/fcvm.2025.1643334

**Published:** 2025-11-13

**Authors:** Shuai Cheng, Bin Yang, Xiaoyu Wang, Pingan Lian, Guangyuan Song, Shenwei Zhang

**Affiliations:** 1 Department of Cardiology, The 7th people’s Hospital of Zhengzhou, Zhengzhou, Henan, China; 2 Henan Provincial Key Laboratory of Cardiac Remodeling and Transplantation, Department of Cardiovascular Surgery, Heart Transplantation Center, The 7th people's Hospital of Zhengzhou, Zhengzhou, Henan, China; 3 Interventional Center of Valvular Heart Disease, National Cardiovascular Disease Clinical Medical Research Center, Beijing Anzhen Hospital, Capital Medical University, Beijing, China; 4 Biotherapy Institute Henan Academy of Innovations in Medical Science, The 7th people’s Hospital of Zhengzhou, Zhengzhou, Henan, China

**Keywords:** left ventricular assist device, aortic regurgitation, transcatheter aortic valve replacement, Taurus Trio valve, femoral artery access

## Abstract

**Background:**

Approximately 25%–30% of patients experience aortic regurgitation (AR) within the first year after left ventricular assist device (LVAD) implantation. However, there is currently no consensus in clinical guidelines regarding the optimal treatment approach for LVAD-associated AR.

**Case summary:**

We report a case of a female patient who developed AR following LVAD implantation. This patient exhibited an open-like configuration of left ventricular outflow tract (LVOT), with no calcified stenosis in the supravalvular region and a lack of anchoring anatomical structures at the junction of the LVOT and sinus-tubular junction. This anatomical configuration posed a high risk of prosthetic valve displacement during conventional transcatheter aortic valve replacement (TAVR). Therefore, we employed a novel TAVR system (Taurus Trio) equipped with a locator, which effectively prevented downward migration of the prosthetic valve after implantation.

**Discussion:**

This case indicates the potential advantages and efficacy of the Taurus Trio valve in TAVR for AR Post-LVAD. We plan to conduct long-term follow-up to further explore and optimize the treatment protocol.

## Introduction

The Left Ventricular Assist Device (LVAD) functions as an alternative treatment for patients with end-stage heart failure awaiting cardiac transplantation (1). By leveraging a catheter to pump blood from the left ventricle into the aorta, the LVAD effectively reduces the workload of the left ventricle and ensures sustained systemic blood perfusion. As a continuous-flow device, the LVAD generates a persistent transvalvular pressure gradient that exerts a retrograde effect on the aortic valve, keeping it in a persistently closed or partially closed state and leading to leaflet fusion, retraction, and degeneration. In addition, the high-velocity jet from the outflow cannula may impose shear stress and mechanical trauma on the valve leaflets. Together, these factors ultimately contribute to the development and progression of aortic regurgitation (AR) (2). Data from previous studies suggest that the incidence of AR within the first year post-LVAD ranges from 25% to 30% (3). Severe AR can disrupt effective LVAD output by creating a closed-loop circulation, exacerbating heart failure and significantly increasing mortality risk.

## Case presentation

### Medical history

**Figure 1:**
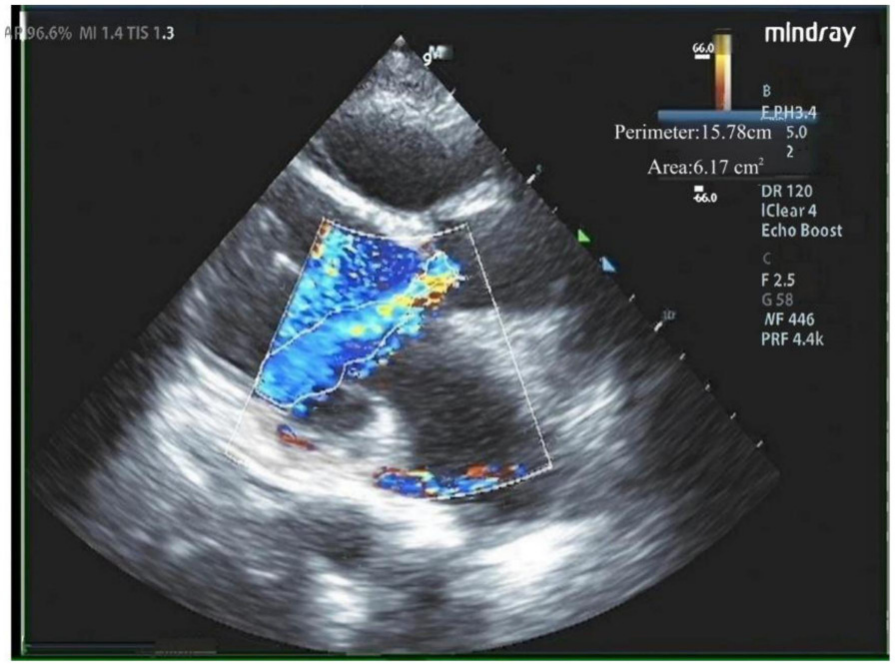
Moderate to severe regurgitation of the aortic valve.

Our hospital admitted a 53-year-old female patient who presented with a chief complaint of “intermittent chest discomfort for 4 years, with exacerbation over the past week”. In 2020, the patient experienced chest discomfort after physical activity, accompanied by shortness of breath and palpitations. She was subsequently diagnosed with ischemic cardiomyopathy and heart failure at a local hospital. Despite receiving guideline-directed medical therapy (GDMT), her condition persisted with recurrent symptoms and progressive worsening. Consequently, on May 8, 2021, she was initiated on continuous renal replacement therapy, utilizing continuous venous-venous hemofiltration. On May 25, 2022, the patient underwent implantation of a LVAD (HeartCon model) in conjunction with coronary artery bypass grafting (CABG). Postoperatively, her symptoms improved significantly, allowing for discharge. However, in June 2024, the patient presented again with chest discomfort and was admitted to our hospital for further evaluation. At admission, the parameters of LVAD were as follows: pump speed 2,300 rpm, power 5.10 W, and flow rate 7.85 L/min. Laboratory tests revealed an elevated N-terminal pro-B-type natriuretic peptide (NT-proBNP) level of 4,864 pg/ml. Echocardiography indicated moderate to severe regurgitation of the aortic valve (Figure 1).

### Determination of treatment protocol

Two years after the implantation of LVAD, the patient exhibited moderate to severe aortic valve insufficiency, as indicated by echocardiography. Additionally, NT-proBNP levels were significantly elevated, suggesting an exacerbation of heart failure symptoms. Despite adjustments to the LVAD flow rate, the therapeutic effect remained suboptimal. This was likely attributed to the AR, which caused blood to flow back into the left ventricle from the ascending aorta due to a persistent pressure gradient. This resulted in a closed-loop circulation that compromised LVAD output and adversely affected its overall functionality. Consequently, addressing the AR became imperative. However, given the patient's history of LVAD implantation and CABG, coupled with severe heart failure, the EuroScore II indicated a high surgical mortality risk of 11.2%. Therefore, the risks associated with performing a conventional surgical aortic valve replacement were considered extremely high. Furthermore, although a dedicated transapical device for AR (such as the J-valve) has been developed and is available in China, for patients with an LVAD implanted at the apex, the apical route is anatomically infeasible due to the presence of the LVAD device. This limitation further supported the decision to select the transfemoral approach in this case. Taking into account this comprehensive evaluation, a transcatheter aortic valve replacement (TAVR) via the femoral artery was proposed as a safer and more viable option.

### Preoperative CT evaluation

**Figure 2:**
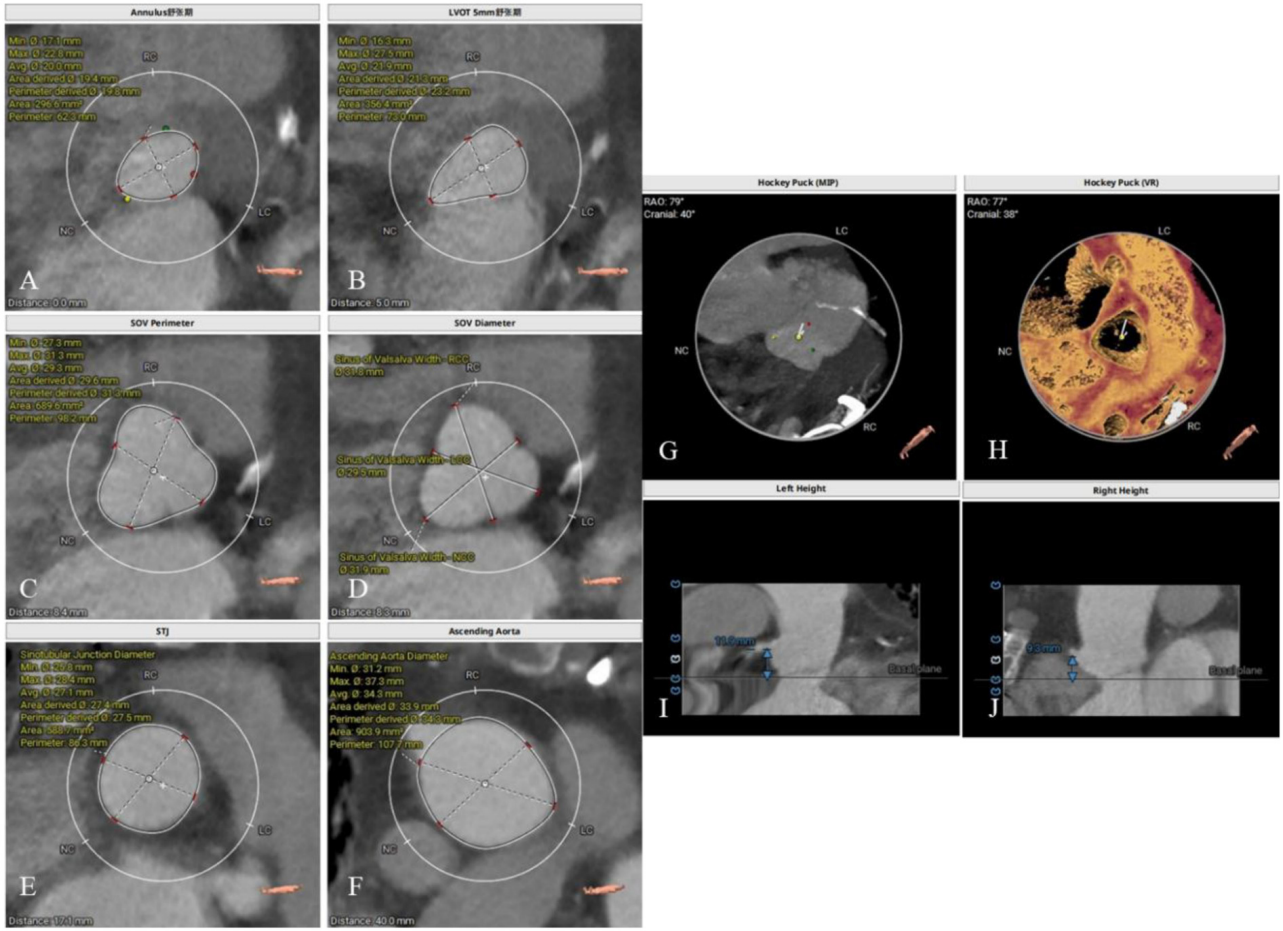
Preoperative CT evaluation. **(A)** Annular circumference diameter: 19.8 mm; **(B)** outflow tract circumference diameter: 23.2 mm; **(C)** sinus of valsalva circumference diameter: 31.3 mm; **(D)** sinus of valsalva diameter: 31.8 mm, 29.5 mm, 31.9 mm; **(E)** sinutubular junction circumference diameter: 27.5 mm; **(F)** aortic circumference diameter 4 cm above the annulus: 34.3 mm; **(G,H)** no significant calcification observed in the annulus and leaflets; **(I)** height of the left coronary artery ostium: 11.9 mm; **(J)** height of the right coronary artery ostium: 9.3 mm.

The patient had a regurgitant tri-leaflet aortic valve with a normally shaped, elliptical-like annulus. The valve leaflets showed no thickening, calcification, or commissural fusion. The annular circumference measured 62.3 mm, with a circumference-derived diameter of 19.8 mm. The diameter of the left ventricular outflow tract (LVOT) exceeded that of the annulus during diastole, giving it an overall funnel-like appearance. The supra-annular region was nearly cylindrical, posing significant challenges for anchoring and elevating the risk of valve migration. Specific measurement data can be found in Figure 2. According to current guidelines, in patients with AR, larger self-expanding valves are generally preferred compared with those used for aortic stenosis, in order to achieve stronger radial force and anchoring. The guidelines recommend an oversizing ratio of prosthetic valve diameter relative to the annular diameter of 15%–30%. The valve selection in this case followed these principles. Based on the preoperative CT evaluation and the absence of significant calcification of the native aortic valve, a Taurus Trio 25 valve (size: 28 mm) was ultimately implanted via the right femoral artery approach to achieve optimal radial support and anchoring stability. The Taurus Trio TAVR system is designed on the basis of the JenaValve Trilogy transcatheter heart valve technology (4). Its unique locator elements enable secure anchoring even in the absence of calcification, effectively preventing valve migration toward the LVOT while ensuring precise coaptation with the native leaflets. This design promotes long-term hemodynamic stability and preserves the possibility of future percutaneous coronary interventions (PCI).

### Surgical procedure

**Figure 3:**
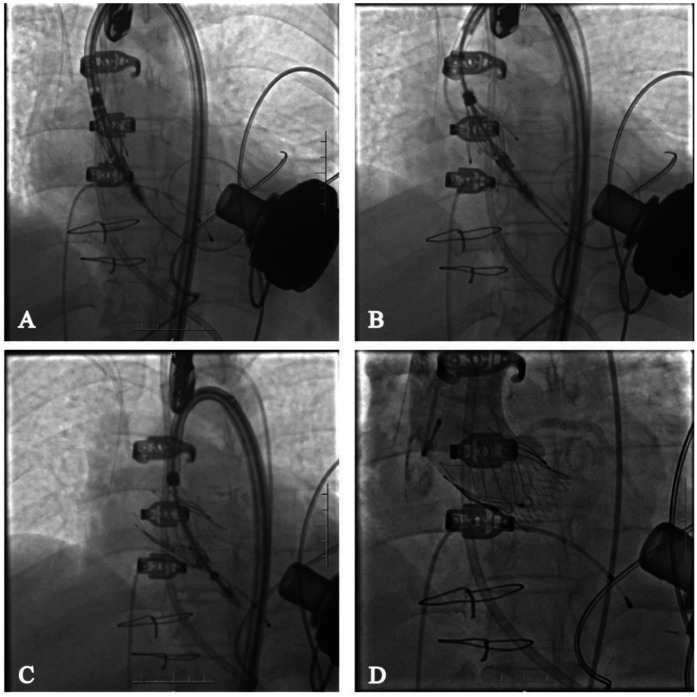
Transcatheter aortic valve replacement via femoral artery. **(A)** Deliver the valve to the annulus location; **(B)** deploy the proprietary locator into the sinus of valsalva; **(C)** release the valve; **(D)** angiography shows no regurgitation of the aortic valve and no impact on coronary perfusion.

**Figure 4:**
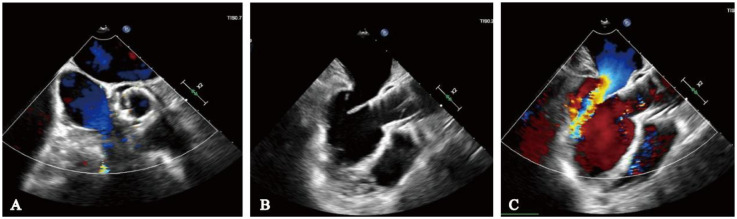
Intraoperative transesophageal echocardiography monitoring. **(A)** No paravalvular leak on short-axis view; **(B)** long-axis view indicates adequate valve depth, no impairment of anterior mitral leaflet function; **(C)** ultrasound color Doppler shows no aortic regurgitation.

The procedure was successfully performed under general anesthesia in the hybrid operating room via the right femoral artery (Figure 3). LVAD pump speed was maintained at 2,250 rpm, consistent with the preoperative setting. A temporary pacing lead was placed through the left femoral vein, and the left femoral artery was used as secondary access. Under fluoroscopic and echocardiographic guidance, a Taurus Trio-THV 25 valve (28 mm) was implanted via the right femoral artery, with all three locators stably anchored at the sinus base. No rapid pacing was required, and the continuous-flow LVAD did not interfere with valve positioning or deployment. Post-deployment aortography demonstrated only trivial paravalvular leak with patent coronary arteries. Intraoperative transesophageal echocardiography confirmed optimal valve position and morphology, with no paravalvular leak and immediate resolution of aortic regurgitation (Figure 4). Given the patient's long-term warfarin therapy (INR 2–3) due to prior LVAD implantation, the main access site was closed with two ProGlide devices, achieving effective hemostasis, and ultrasound confirmed the absence of bleeding, dissection, or stenosis.

### Follow-up

**Figure 5:**
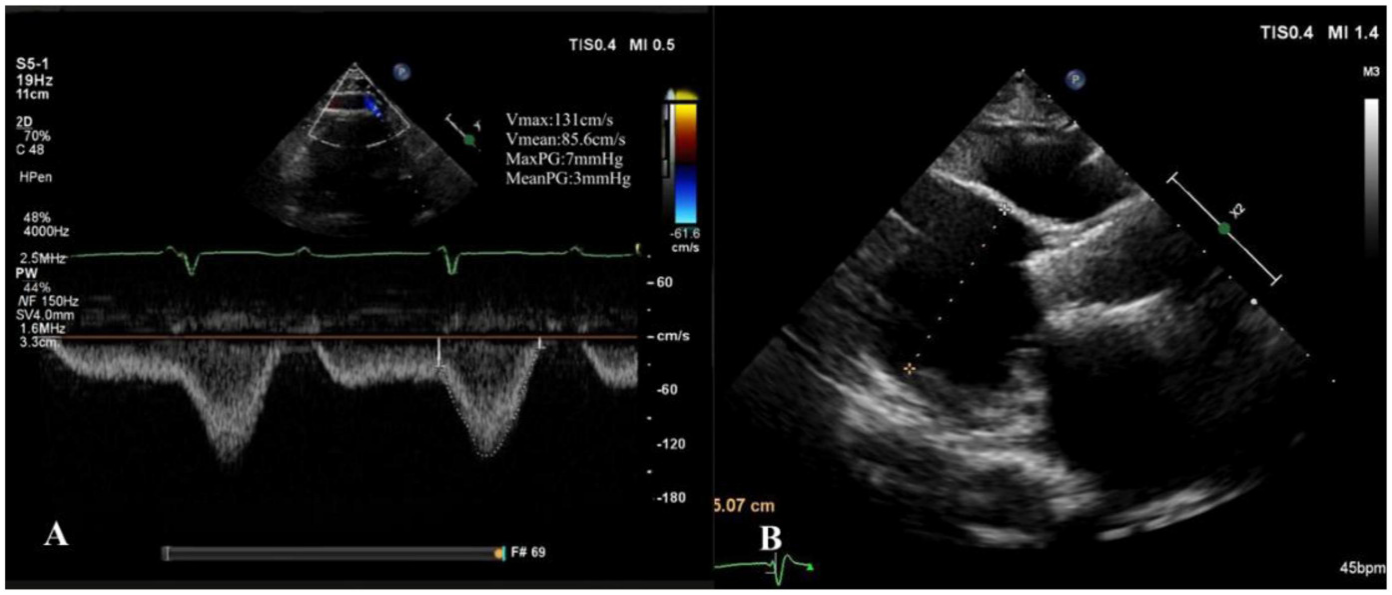
One-month postoperative echocardiographic follow-up. **(A)** Spectral Doppler showing no evidence of aortic regurgitation; **(B)** long-axis view demonstrating no late prosthetic valve migration.

At the 1-month follow-up, the patient's symptoms had markedly improved (NYHA functional class II). Transthoracic echocardiography demonstrated normal morphology and function of the prosthetic valve, with no evidence of late prosthesis migration, and spectral Doppler imaging revealed no regurgitation (Figure 5). LVAD parameters remained stable throughout the follow-up period (pump speed 2,200–2,300 rpm, flow approximately 2.5 L/min).

## Discussion

Heart failure represents the common final pathway of various cardiac diseases and remains a growing global health burden despite advances in primary prevention strategies (5). For patients with end-stage disease refractory to guideline-directed medical therapy (GDMT), cardiac transplantation is the gold standard; however, limited donor availability allows fewer than 10% of eligible patients to undergo transplantation (6). Against this background, left ventricular assist devices (LVADs) have emerged as a crucial alternative, either as a bridge to transplant or as destination therapy (7). With the advent of third-generation continuous-flow LVADs, mid-term survival has approached that of heart transplantation, highlighting their increasing clinical potential (8).

Nevertheless, the hemodynamic characteristics of continuous-flow LVADs predispose to unique complications. Because blood is continuously diverted from the left ventricle into the ascending aorta via the outflow graft, the aortic valve remains closed or minimally opens. This altered physiology impairs valve function and promotes the development of aortic regurgitation (AR) (9). Mechanistically, persistent transvalvular pressure gradients, increased retrograde flow, and LV unloading create a “closed loop” circulation with blood repeatedly regurgitating into the left ventricle (10). This phenomenon attenuates the unloading effect of LVAD support, precipitates recurrent heart failure, and has been associated with increased morbidity and mortality (11).

Accordingly, post-LVAD AR represents a critical challenge to long-term LVAD therapy, yet therapeutic strategies remain limited. The initial management of symptomatic AR typically consists of medical therapy, including diuretics, vasodilators, and LVAD speed reduction (12). However, in cases refractory to medical treatment, surgical aortic valve replacement or heart transplantation remains the primary therapeutic option. In LVAD recipients, repeat sternotomy is often associated with high procedural risk, and not all patients are suitable candidates for transplantation (13, 14). The use of the Amplatzer septal occluder (Abbott) to close the aortic valve has been investigated, but this approach is limited by high rates of AR recurrence (15, 16). Moreover, complete aortic valve closure renders patients fully dependent on LVAD support for systemic perfusion, and any device dysfunction can rapidly become fatal (15, 16).

Transcatheter aortic valve replacement (TAVR), initially developed for severe aortic stenosis, has been increasingly applied to AR, including in LVAD recipients (17). However, several unique challenges arise in the post-LVAD setting. Anatomical considerations include annular dilatation, enlarged left ventricular outflow tract (LVOT), and the absence of calcific anchoring, which compromise secure valve positioning. Registry data of off-label TAVR with conventional devices such as CoreValve and Sapien have demonstrated higher rates of valve embolization, paravalvular leak, and frequent need for a second valve compared with procedures for aortic stenosis (18, 19). Hemodynamic considerations are equally significant: continuous forward flow and increased retrograde flow caused by the LVAD create a complex environment that hinders device stabilization and may necessitate intraoperative adjustments such as pump speed modulation or brief pump pauses to optimize deployment (20, 21).

In recent years, valve systems specifically designed for AR have been developed. The first-generation transapical JenaValve achieved procedural success rates of approximately 97%–100% in non-calcified AR, but valve migration and reintervention remained concerns (22–24). The next-generation transfemoral JenaValve Trilogy, employing a leaflet-locating mechanism for anchoring independent of calcification, demonstrated a 95% technical success rate in the ALIGN-AR trial, with a 30-day composite safety endpoint of 27% and a 1-year all-cause mortality of 7.8%, supporting its favorable safety and efficacy in early follow-up (25).

In addition, China has been actively developing transcatheter valve systems specifically designed for AR. The TaurusTrio (licensed from the Trilogy system by Peijia Medical) has entered pivotal clinical trials at multiple centers and achieved its first successful implantation in 2023, demonstrating favorable early feasibility and potential stability. In the present case, we selected the TaurusTrio system with dedicated locators for TAVR. Its unique design enabled stable positioning and deployment even in the absence of annular calcification and under continuous LVAD flow. Postoperative imaging follow-up confirmed satisfactory valve function without migration or regurgitation, and the patient experienced marked symptomatic improvement, with NYHA functional class improving to II at one month. This case suggests that dedicated AR valves may represent an optimal therapeutic option for patients with LVAD-associated AR and could provide important insights for future clinical practice and guideline development.

## Data Availability

The original contributions presented in the study are included in the article/Supplementary Material, further inquiries can be directed to the corresponding authors.
